# Discordance between antral follicle counts and anti-Müllerian hormone levels in women undergoing in vitro fertilization

**DOI:** 10.1186/s12958-019-0497-4

**Published:** 2019-07-04

**Authors:** Yangyang Zhang, Yang Xu, Qing Xue, Jing Shang, Xiuli Yang, Xuemin Shan, Yanrong Kuai, Sheng Wang, Cheng Zeng

**Affiliations:** Department of Obstetrics & Gynecology, Peking University First Hospital, Beijing, 100034 China

**Keywords:** Antral follicle count, Anti-Müllerian hormone, Ovarian response, Clinical pregnancy rate

## Abstract

**Background:**

In general, anti-Müllerian hormone (AMH) is positively associated with antral follicle count (AFC). However, there is often discordance between the AMH level and AFC in clinical practice. In cases of discordance, which indicator should be chosen to predict ovarian response and subsequently develop an ovulation induction protocol? The objective of this study was to investigate which indicator was more accurate in predicting ovarian response and pregnancy outcomes when the AMH level and AFC were discordant.

**Methods:**

A total of 1121 infertile women undergoing IVF/ICSI were recruited in this study. During the study period, patients were subjected to individualized controlled ovarian hyperstimulation (COH) protocols according to specific characteristics. The AMH levels and AFCs were measured on days 2–3 of the menstrual cycle. Serum samples were obtained to determine AMH levels. Transvaginal ultrasound was performed to determine the AFC. All patients were divided into four groups: Group A had AFCs and AMH levels in the normal range; Group B had normal AFCs and low AMH levels; Group C had low AFCs and normal AMH levels; and Group D had low AFCs and AMH levels.

**Results:**

Two hundred three women (18.11%) showed discordant AFCs and AMH levels. In the two groups with discordant AFCs and AMH levels, namely, Group B and Group C, the oocyte yield, good-quality embryo rate and clinical pregnancy rate were significantly higher in Group B than in Group C. The incidence of poor ovarian response (POR) was significantly lower in Group B than in Group C. According to the stratified analysis of age, for the three categories above the age of 30, oocyte yield was higher in Group B than in Group C. In all age categories, the clinical pregnancy rate was higher in Group B than in Group C.

**Conclusions:**

Our study demonstrated that approximately one in five patients in clinical practice showed discordance between AFCs and AMH levels. In view of the AFC being better than AMH for predicting POR, the AFC should be the preferred indicator for predicting ovarian response to subsequently develop an optimal individualized COH protocol.

## Background

In the process of controlled ovarian hyperstimulation (COH), the key steps are to evaluate ovarian reserve function, predict ovarian response, and develop an optimal individualized COH protocol. Only in this way, it is possible to obtain an appropriate quantity of oocytes and attain high-quality oocytes, and ultimately produce a sufficient number of high-quality embryos and improve the clinical pregnancy rate. There are many indicators for predicting ovarian response and pregnancy outcomes, including age, hormone levels (basal follicle stimulating hormone (FSH), oestrogen (E_2_), anti-Müllerian hormone (AMH), etc.) and ultrasound indicators (ovarian volume, antral follicle count(AFC), etc.) [1]. Current studies have shown that the AMH level and AFC have higher predictive value for poor ovarian response (POR) than other indicators [2, 3], and the accuracy of prediction is consistent [4].AMH is produced by the granulosa cells of pre-antral and small antral follicles, and its level is not affected by the menstrual cycle [5] or exogenous hormonal supplementation [6].Therefore, AMH levels can better represent the number of primordial follicles and reflect ovarian reserve function. The AFC refers to the number of follicles with diameters of 2 mm to 9 mm [7]; these follicles begin to develop after recruitment in the luteal phase of the previous cycle and generally reflect the number of follicles that will continue to mature during the ovulation treatment cycle. In general, AMH is positively associated with AFC; that is, patients with good ovarian reserve function have high AMH and AFC values, and those with poor ovarian reserve function have low values. However, there is often discordance between the AMH level and AFC in clinical practice. In cases of discordance, which indicator should be chosen to predict ovarian response and subsequently develop an ovulation induction protocol? The objective of this study was to investigate which indicator was more accurate in predicting ovarian response and pregnancy outcomes when the AMH level and AFC were discordant.

## Methods

### Patients

A total of 1121 infertile women undergoing in vitro fertilization (IVF) /intracytoplasmic sperm injection (ICSI) were recruited in this study from January 2016 to December 2017 at the Reproductive and Genetic Medical Center of Peking University First Hospital. Women with a history of ovarian surgery, polycystic ovarian syndrome (PCOS), hormonal therapy in the past 6 months or other endocrine diseases, including diabetes mellitus, thyroid disease, and hyperprolactinemia were excluded from this study. This study was approved by the Clinical Research Institutional Review Board of Peking University First Hospital, and all patients provided informed consent.

### COH protocols

During the study period, each patient was subjected to an individualized COH protocol according to specific characteristics, such as ovarian reserve and follicle size. Gonadotropin therapy was performed, and follicles were regularly monitored by transvaginal ultrasound. Recombinant human chorionic gonadotropin (HCG) was administered subcutaneously when the leading follicle was 18–20 mm in diameter. Oocytes were retrieved by transvaginal ultrasound-guided follicular aspiration within approximately 36 h after HCG administration. Oocytes were fertilized by conventional IVF/ICSI, and embryos were transferred under abdominal ultrasound guidance on day 3 after oocyte retrieval. HCG tests were performed on day 14 after ET, and if the result was positive, luteal support was continued as before until 10 weeks of gestation. Clinical pregnancy was defined as the presence of an intrauterine gestational sac 4 weeks after ET. The transplantation was cancelled for the following reasons: (1)to prevent the occurrence of ovarian hyperstimulation syndrome (OHSS); (2) when no transplantable embryos were obtained; (3) to accumulate embryos; or (4) when progesterone levels were > 2.5 ng/ml on HCG day.

Patients were divided into four groups according to the boundaries for the AFC and AMH level in the ovarian reserve test provided by the “Bologna criteria” [8]:

- Group A, AFC≥7 and AMH≥1.1 ng/ml, 611 women (both AFCs and AMH levels in the normal range);
- Group B, AFC≥7 and AMH<1.1 ng/ml, 85 women (normal AFCs and low AMH levels);
- Group C, AFC<7 and AMH≥1.1 ng/ml, 118 women (low AFCs and normal AMH levels);
- Group D, AFC<7 and AMH<1.1 ng/ml, 307 women (low AFCs and low AMH levels).

The following criteria were used to define ovarian response according to oocyte yield [9]: POR, oocyte yield< 4; normal ovarian response, oocyte yield≥4 and ≤ 15; and high ovarian response, oocyte yield> 15.

### Assessment of AMH levels and AFCs

Serum samples were obtained on days 2–3 of the spontaneous menstrual cycle prior to one month of IVF/ICSI treatment in agonist protocol cycles for serum AMH measurements. In antagonist cycles, serum samples were obtained on days 2–3 of the treatment cycle. Serum samples were separated within 1 h after collection and stored at − 80 °C until analysis for AMH. Serum AMH levels were analysed by enzyme-linked immunosorbent assay (ELISA).

Transvaginal ultrasound was performed to assess the AFC on days 2–3 of the treatment cycle using an Aloka SSD-1000 (Japan) with a 5 MHz transvaginal probe by one of four doctors for each case. Operators underwent uniform training prior to the start of the study to reduce errors caused by different operators. Follicles measuring 2 mm–10 mm in diameter were counted in both ovaries to determine the AFC. The total number of follicles in both ovaries was used as the AFC.

### Statistical analysis

All analyses were performed with the Software Package for Social Sciences (SPSS) version 13.0 for Windows. All normal distribution measurement data are expressed as the mean ± standard deviation (SD). Comparisons between two groups were analysed with independent sample t-tests, comparisons among multiple samples were analysed by variance analysis, and intergroup multiple comparisons were analysed with the Bonferroni correction. The count data were analysed by chi-square test. *P* < 0.05 was considered statistically significant.

## Results

In this study, the AFCs and AMH levels of 918 women (81.89%) were concordant in that they were both either normal or low. The AFCs and AMH levels of 203 women (18.11%) were discordant. Eighty-five women (7.58%) had normal AFC values and low AMH values (Group B), and 118 women (10.53%) had low AFC values and normal AMH values (Group C). Table 1 summarizes the characteristics of the groups.

**Table 1:** Comparison of clinical data of patients in different groups

Parameters	Group A (*n* = 611)	Group B (*n* = 85)	Group C (*n* = 118)	Group D (*n* = 307)	*p* value
Age (years)	32.69 ± 4.59	33.98 ± 4.89▲	36.81 ± 4.79▲	37.74 ± 5.16	< 0.001
Basal FSH (mIU/mL)	7.48 ± 2.20	8.81 ± 2.87▲	10.14 ± 4.57▲	11.24 ± 4.83	< 0.001
No. of AFCs (n)	12.29 ± 4.08	8.47 ± 1.72▲	4.53 ± 1.41▲	3.69 ± 1.59	< 0.001
AMH level (ng/ml)	3.46 ± 1.88	0.76 ± 0.24▲	1.94 ± 1.03▲	0.51 ± 0.30	< 0.001
Oocyte yield (n)	10.63 ± 5.14	5.13 ± 2.98▲	4.11 ± 2.50▲	2.10 ± 1.78	< 0.001
Poor ovarian response (%)	5.07%(31/611)	25.88%▲(22/85)	40.68%▲(48/118)	83.06%(255/307)	< 0.001
Fertilization rate (%)	72.41%(4702/6494)	73.85%(322/436)	73.20%(355/485)	73.37%(474/646)	0.862
Good-quality embryo rate (%)	41.01%(1961/4782)	45.51%▲ (152/334)	35.28%▲ (145/411)	25.21%(121/480)	0.004
Transplant cancellation rate (%)★	33.88%(207/611)	32.94%(28/85)	46.61%(55/118)	68.73%(211/307)	< 0.001
Clinical pregnancy rate (%)	43.32% (175/404)	43.86%▲(25/57)	23.81%▲(15/63)	25.00%(24/96)	< 0.001

▲Group B and Group C have significant differences. (*P* < 0.05)

★ The main reasons for transplant cancellation in the four groups were different

Group A: to prevent the occurrence of OHSS (55.56%, 115/207)

Group B: no transplantable embryos (32.14%, 9/28) and to accumulate embryos (32.14%, 9/28)

Group C: no transplantable embryos (29.09%, 16/55) and to accumulate embryos (23.64%, 13/55)

Group D: no transplantable embryos (52.61%, 111/211) and to accumulate embryos (28.44%, 60/211)

As shown in Table 1, for all study subjects, patient age (*p* < 0.001), FSH level (p < 0.001) and POR rate (p < 0.001) progressively increased, and oocyte yield (p < 0.001) progressively decreased from Group A to Group B to Group C and to Group D. The clinical pregnancy rates of Group A and Group B were significantly higher than those of Group C and Group D.

In the two groups with discordant AFCs and AMH levels, namely, Group B (normal AFCs and low AMH levels) and Group C (low AFC and normal AMH levels), the oocyte yield, good-quality embryo rate and clinical pregnancy rate were significantly higher in Group B than in Group C. The incidence of POR in Group B was significantly lower than that in Group C.

**Fig. 1:**
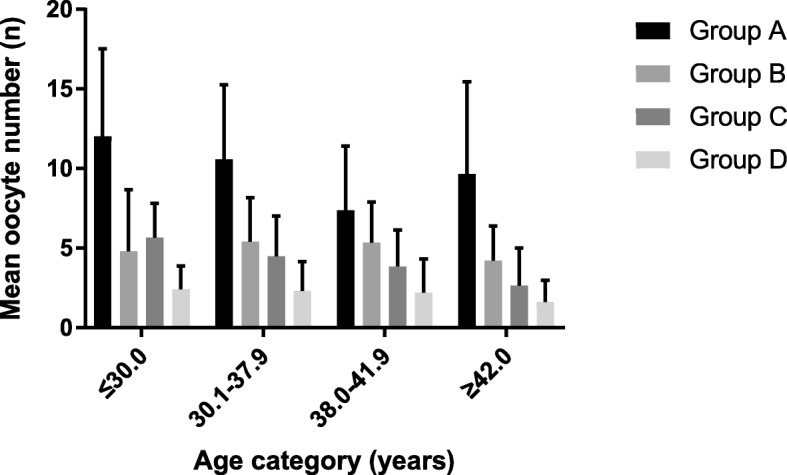
Mean oocyte number in all age categories. Among all age categories, oocyte yield was highest in Group A and lowest in Group D. Although there were no statistically significant differences in oocyte yield between Group B and Group C, oocyte yield was higher in Group B than in Group C in all age categories except for the ≤30.0age category

Patients were stratified into the following age categories: ≤30.0, 30.1–37.9, 38.0–41.9, and ≥ 42.09 years. As shown in Table 2, the incidences of AMH and AFC discordancy in the four age categorieswere12.72% (36/283), 17.79% (87/489), 23.83% (51/214), and 21.48% (29/135), respectively. There were statistically significant differences. Figure 1 showed that oocyte yield was highest in Group A and lowest in Group D among all age categories. Although there were no statistically significant differences in oocyte yield between Group B and Group C, oocyte yield was higher in Group B than in Group C in all age categories except for the ≤30.0age category. In all age groups, the clinical pregnancy rate of Group B was higher than that of Group C, and there were significant differences in the 38.0–41.9 age categories.

**Table 2:** Clinical data of patients in different age categories

age	Group	N	AFC (n)	AMH (ng/ml)	Oocyte yield (n)	Clinical pregnancy rate (%)
≤30.0	A	217	13.39 ± 4.15	3.83 ± 2.06	12.02 ± 5.51	44.68%(63/141)
(*n* = 283)	B	21	8.52 ± 1.81	0.78 ± 0.27	4.81 ± 3.87	33.33%(4/12)
	C	15	5.27 ± 1.28	2.30 ± 1.31	5.67 ± 2.16	22.22%(2/9)
	D	30	3.97 ± 1.73	0.54 ± 0.24	2.43 ± 1.46	26.67%(4/15)
30.1–37.9	A	293	12.11 ± 3.99	3.39 ± 1.81	10.58 ± 4.67	45.78%(92/201)
(*n* = 489)	B	44	8.50 ± 1.80	0.73 ± 0.25	5.41 ± 2.77	47.06%(16/34)
	C	43	4.47 ± 1.50	1.89 ± 0.62	4.49 ± 2.53	36%(9/25)
	D	109	3.88 ± 1.40	0.58 ± 0.30	2.32 ± 1.84	32.5%(13/40)
38.0–41.9	A	83	10.16 ± 3.37	2.89 ± 1.52	7.39 ± 4.02	31.48%(17/54)
(*n* = 214)	B	11	8.18 ± 1.47	0.77 ± 0.19	5.36 ± 2.54	66.67%(4/6)
	C	40	4.43 ± 1.36	2.12 ± 1.34	3.85 ± 2.29	15.00%(3/20)
	D	80	3.80 ± 1.62	0.49 ± 0.31	2.21 ± 2.11	27.27%(6/22)
≥42.0	A	18	11.78 ± 3.58	2.53 ± 0.88	9.67 ± 5.79	37.50%(3/8)
(*n* = 135)	B	9	8.56 ± 1.67	0.86 ± 0.11	4.22 ± 2.17	20.00%(1/5)
	C	20	4.35 ± 1.31	1.43 ± 0.51	2.65 ± 2.37	11.11%(1/9)
	D	88	3.27 ± 1.69	0.43 ± 0.29	1.63 ± 1.36	5.26%(1/19)

## Discussion

There are many indicators for predicting ovarian response. In general, with advancing age, FSH levels increase, and AMH levels and AFC decrease. In the course of clinical diagnosis and treatment, there are often discordances in relevant indicators. Previous studies have examined the concordance of FSH and AMH levels. Gleicher et al. investigated discordances between FSH and AMH levels in366 consecutive infertility patients. They found that oocyte yield declined in the following order: normal AMH and FSH levels, normal AMH but abnormal FSH levels, abnormal AMH but normal FSH levels, and abnormal AMH and FSH levels. Therefore, they demonstrated that a normal AMH level is more important than a normal FSH level [10]. Leader et al. measured the AMH and FSH levels of 5354 infertile women from days 2 to 4 of the menstrual cycle and found that one in five women had discordant AMH and FSH values [11]. The AMH level and AFC are the two most accurate indicators for predicting ovarian response [7]. Discordances between them have not been reported in the literature.

In general, serum AMH levels are strongly positively correlated with AFCs [12]. Li et al. retrospectively studied 1156 women undergoing their first IVF cycle, and the results demonstrated that AMH level was significantly correlated with AFC. Both AMH and AFC showed significant correlations with age and ovarian response [13]. However, there are often discordances between the AMH level and AFC in clinical practice. In cases of discordance, which indicator should doctors choose to predict ovarian response and subsequently develop an ovulation induction protocol? Our results demonstrated that approximately one in five infertile women had discordance in the AFC and AMH level. The oocyte yield and clinical pregnancy rate were significantly higher in Group B (normal AFCs and low AMH levels) than in Group C (low AFC and normal AMH). The incidence of POR in Group B was significantly lower than that in Group C. According to the stratified analysis of age, for the three categories above the age of 30, oocyte yield was higher in Group B than in Group C. In all age categories, the clinical pregnancy rate of Group B was higher than that of Group C. The results suggest that a normal AFC is more important than a normal AMH value in predicting ovarian response. In other words, once the AFC becomes abnormal, even if the AMH level is still normal (Group C), the oocyte yield decreases. Therefore, we suggest that the AFC should be prioritized in the predicting of ovarian response and the clinical pregnancy rate in cases of discordance between the AFC and AMH level.

Why is there discordance between the AFC and AMH level? We speculate that this may be due to differences in the follicle population represented by the two indicators. The AFC refers to the number of follicles with diameters of 2 mm to 9 mm. These follicles are mostly gonadotropin responsive and selectable for further growth and development through to the preovulatory stage during COH. Therefore, the AFC is a direct marker of the recruitable follicular cohort [7]. AMH is a dimeric glycoprotein of the transforming growth factor-b (TGF-b) family. AMH is produced by granulosa cells of pre-antral and small antral follicles of less than 4 mm diameter in the ovary [14]. AMH indirectly reflects the population of early growing follicles [15]. These small follicles are not able to enter the follicular recruitment stage during the ovulation induction cycle, let alone develop into mature follicles for egg retrieval and fertilization. Therefore, the AMH level mainly reflects the ovarian reserve function [16].

The female fertility decreases with increasing chronological age has been known for a long time. This age-related decline is most likely due to a gradual decline in both oocyte quantity and quality [17, 18]. According to the stratified analysis of age, in the category above the age of 42, oocyte yield in Group A (AFCs and AMH levels in the normal range) and Group B (normal AFCs and low AMH levels) showed no significant reduction compared with that in the other age groups, and clinical pregnancy rates reached 37.50 and 20.00%, respectively. Therefore, we suggest that for older infertile women, as long as the AFC is normal, IVF treatment should be actively performed, even if the AMH level is low.

## Conclusions

In conclusion, the AFC and AMH level are the two most accurate indicators for predicting ovarian response and pregnancy outcomes. Our study demonstrated that approximately one in five patients in clinical practice had discordance in their AFCs and AMH levels. In view of the AFC being better than the AMH level for predicting POR, the AFC should be preferred in the prediction of ovarian response, to ultimately develop an optimal individualized COH protocol.

## Data Availability

The datasets used and/or analysed during the current study are available from the corresponding author on reasonable request.

## Abbreviations

AFC: Antral follicle count
AMH: Anti-Müllerian hormone
COH: Controlled ovarian hyperstimulation
E_2_: Oestrogen
ELISA: Enzyme-linked immunosorbent assay
FSH: Follicle stimulating hormone
HCG: Human chorionic gonadotropin
ICSI: Intracytoplasmic sperm injection
IVF: In vitro fertilization
OHSS: Ovarian hyperstimulation syndrome
PCOS: Polycystic ovarian syndrome
POR: Poor ovarian response
SD: Standard deviation
SPSS: Software Package for Social Sciences
